# Fabrication and Characterization of Electrospun Poly(acrylonitrile-*co*-Methyl Acrylate)/Lignin Nanofibers: Effects of Lignin Type and Total Polymer Concentration

**DOI:** 10.3390/polym13070992

**Published:** 2021-03-24

**Authors:** Suchitha Devadas, Saja M. Nabat Al-Ajrash, Donald A. Klosterman, Kenya M. Crosson, Garry S. Crosson, Erick S. Vasquez

**Affiliations:** 1 Department of Chemical and Materials Engineering, University of Dayton, Dayton, OH 45469, USA; devadass1@udayton.edu (S.D.); nabats1@udayton.edu (S.M.N.A.-A.); dklosterman1@udayton.edu (D.A.K.); 2 Department of Civil and Environmental Engineering and Engineering Mechanics, University of Dayton, Dayton, OH 45469, USA; kcrosson1@udayton.edu; 3 Integrative Science and Engineering Center, University of Dayton, Dayton, OH 45469, USA; 4 Department of Chemistry, University of Dayton, Dayton, OH 45469, USA; gcrosson1@udayton.edu

**Keywords:** electrospinning, alkali, kraft lignin, low sulfonate lignin, poly(acrylonitrile-*co*-methyl acrylate), nanofibers, modulated DSC, lignin-based nanofibers

## Abstract

Lignin macromolecules are potential precursor materials for producing electrospun nanofibers for composite applications. However, little is known about the effect of lignin type and blend ratios with synthetic polymers. This study analyzed blends of poly(acrylonitrile-*co*-methyl acrylate) (PAN-MA) with two types of commercially available lignin, low sulfonate (LSL) and alkali, kraft lignin (AL), in DMF solvent. The electrospinning and polymer blend solution conditions were optimized to produce thermally stable, smooth lignin-based nanofibers with total polymer content of up to 20 wt % in solution and a 50/50 blend weight ratio. Microscopy studies revealed that AL blends possess good solubility, miscibility, and dispersibility compared to LSL blends. Despite the lignin content or type, rheological studies demonstrated that PAN-MA concentration in solution dictated the blend’s viscosity. Smooth electrospun nanofibers were fabricated using AL depending upon the total polymer content and blend ratio. AL’s addition to PAN-MA did not affect the glass transition or degradation temperatures of the nanofibers compared to neat PAN-MA. We confirmed the presence of each lignin type within PAN-MA nanofibers through infrared spectroscopy. PAN-MA/AL nanofibers possessed similar morphological and thermal properties as PAN-MA; thus, these lignin-based nanofibers can replace PAN in future applications, including production of carbon fibers and supercapacitors.

## 1. Introduction

Many of today’s leading industries—such as aerospace, automobile, military, and energy—are increasingly pursuing sustainable materials with enhanced properties, including low density, good thermal stability, corrosion resistance, and high stiffness and strength. These performance enhancements traditionally have been achieved by using composite materials, most notably carbon fiber composites, offering an alternative to denser and more expensive metallic and ceramic material [1,2]. Commercially available precursor materials for carbon fiber production are synthesized from petroleum feedstocks, such as polyacrylonitrile (PAN) [3,4,5]. Recently, natural polymers such as cellulose, curdlan, and lignin emerged as alternatives for preparing composite materials due to their wide availability, cost-effectiveness, and recyclability [6,7,8,9]. For instance, lignin is a traditional by-product of the paper and pulp industry with high carbon content and multiple aromatic structures. In addition to these attributes, lignin macromolecules can exhibit hydrophilic or hydrophobic properties and are used as UV-protecting materials [6,10]. Hence, lignin has emerged as a potential sustainable precursor for fabricating carbon-based fibers, composites, or nanomaterials products. However, due to its low molecular weight, lignin usage is limited and is currently used only as an additive material or filler in synthetic polymers. Despite this limitation, partially bio-based carbon nanofibers made from blends of lignin macromolecules and other polymers such as PAN are cost-effective and could be used as value-added products [11,12]. For example, alkali lignin/PAN blends exhibited higher elastic modulus and tensile strength than pure PAN [13]. These enhanced properties could allow the fabrication and design of improved membranes, adsorbents, or electrical materials [14].

As recently demonstrated through scale-up processes, electrospinning has emerged as a viable tool in the manufacturing of natural nanocomposite fibers [15,16,17,18]. In the electrospinning process, a high electrical field is applied to obtain a jet flow of polymer solution by rapid solvent evaporation, resulting in the formation of microfibers or nanofibers [19,20]. Conditions such as polymer concentration, molecular weight of polymers, viscosity, electrical conductivity, and surface tension affect nanofiber formation [21,22]. Additionally, electrospinning has enabled the fabrication of nanofibers comprised of polymer blends, including natural polymers such as lignin [23,24]. These exhibit high porosity, higher electrical conductivity, improved mechanical properties, and high surface area compared to the neat polymer. For example, biorefinery lignin/PAN based carbon fibers enhanced via graphene modification used in electrodes improved electrical conductivity and quantification of acetaminophen [25]. Electrospun organosolv lignin/PAN nanofibers flexibility and thermochemical properties were improved at higher lignin contents [26]. Adsorptive removal of Pb(II) contaminants from wastewater was successful using sago-lignin/PAN electrospun carbon nanofibers [27]. In addition to PAN, lignin blends with other polymer matrices such as polyvinyl alcohol (PVA) or polyethylene oxide (PEO) exhibited enhanced properties [28,29,30]. For example, phosphoric acid modified kraft lignin has been shown to increase the mechanical properties of electrospun lignin/PEO fibers due to an increase in lignin’s molecular weight [31].

The rheological, electrical conductivity and surface tension of polymer solutions heavily influence the morphological properties of electrospun nanofibers [32]. For instance, the rheological properties of low molecular weight organosolv lignin/PAN polymer solutions were analyzed over a range of polymer concentrations and blend ratios to optimize the fabrication of defect-free, smooth nanofibers [33]. Electrospun nanofibers comprised of alkali lignin/PVA and PAN had larger pore sizes compared to traditional activated carbon, and the alkali lignin/PVA nanofibers demonstrated enhanced and faster adsorption of a large molecule adsorbate [34]. Dallmeyer et al. [35] examined the physical properties and chemical structure differences of electrospun kraft lignin that influence shape change and recovery, as well as moisture responses of fractions of lignin. Cho et al. [36] analyzed functional groups on solvent cast film and nanofibers using Fourier transform infrared spectroscopy (FTIR) and polarized light optical microscopy analysis in order to understand alignment of lignin within the structured films. They also analyzed the birefringence of lignin nanofibers under changing angles of polarized light.

PAN homopolymers, blended with lignin, are the traditional precursor materials for the preparation of carbon nanofibers through electrospinning as discussed recently [23]; however, PAN lacks drawability and possess poor heat treatment tolerance due to the interactions among nitrile groups [37]. As a result, PAN copolymerization with methyl acrylate (MA), vinyl acetate (VAc) or itaconic acid (IA) has been used to enhance nanofiber spinnability, as well as thermal performance [38]. Although lignin cannot be electrospun directly due to its molecular weight branched structure and lack of chain entanglement, PAN-*co*-MA copolymer blended with lignin fillers could produce stable nanofibers. Furthermore, the quality and spinnability of nanofibers are also governed by lignin type. Lignins are classified by their extraction methods and also on the plant resource such as organosolv lignin, alkali, Kraft lignin, and lignosulfonates.

The current study intends to shed light on the potential for producing thermally stable, uniform nanofibers from blends of the most common types of commercially available lignin, i.e., low sulfonate lignin (LSL) and alkali, kraft lignin (AL), with the copolymer PAN-*co*-MA rather than with pure PAN. We prepared PAN-*co*-MA/lignin blends at different weight ratios (50/50 and 80/20) and total polymer content (10%, 12%, and 20%) in DMF solvents for each lignin type. Using these polymers and blends, for the first time, we investigated the correlations between the polymer solution behavior (i.e., lignin distribution within PAN-*co*-MA/lignin solvent cast films and polymer solution rheological studies) with the fabricated electrospun nanofibers morphological, thermal, and chemical properties. Furthermore, we discussed the effects of lignin sulfonation by using LSL and AL in the preparation and characterization of lignin-based electrospun nanofibers through microscopy, thermal, and spectroscopy analyses.

## 2. Materials and Methods

### 2.1. Materials

Alkali Lignin, low sulfonate content (LSL), alkali, kraft lignin (AL), and N,N-Dimethylformamide (DMF) anhydrous, 99.8% were purchased from Sigma-Aldrich (Saint Louis, MO, USA). Poly (acrylonitrile-*co*-methyl acrylate) with acrylonitrile ~94 wt %, MW 100,000 was procured from Scientific Polymer Products, Inc. (Ontario, NY, USA). Hereafter, the copolymer is referred to as PAN-MA. Chemicals were used as received, without further purification.

### 2.2. Preparation of Polymer Blends and Electrospinning Parameters

PAN-MA was initially dissolved in DMF at 60 °C and mixed in 20 mL scintillation vials with magnetic stirring at 600 rpm for 30 min. Next, specific masses of LSL or AL powder were added to the PAN-MA solution and magnetically stirred. PAN-MA/LSL mixtures were stirred for 12 h, whereas PAN-MA/AL solutions were mixed for 2 h due to greater solubility. Table 1 shows the specific lignin types and concentration used for each blend. Three types of electrospun nanofibers were prepared using these polymer solutions: PAN-MA (control), PAN-MA/LSL, and PAN-MA/AL. Based on the PAN-MA/AL solubility in DMF, the total polymer content, including both PAN-MA and lignin, varied from concentrations of 10, 12, and 20 wt % (Table 1).

Each prepared polymer blend was filled transferred a 10 mL plastic syringe with a blunt 18G needle (~1 mm inner diameter) and mounted on a syringe pump (New Era Pump Systems, Inc., NE-300, Farmingdale, NY, USA). Electrospinning was conducted at a 0.003 mL/min flow rate. A voltage generator (Stanford Research Systems, Inc. model P.S375, Sunnyvale, CA, USA) was operated at 15 kV using a positive voltage supplied to the needle. A negative voltage (6–8 volts) was used to control a rotatory cylindrical drum collector (Dayton DC Motor, OH, USA 4Z145). Electrospun nanofibers were collected on aluminum foil. The working distance between the collector and the needle was fixed at 21 cm. Fabricated nanofibers were heat-treated in a vacuum oven (Fisher Isotemp^®^ standard capacity) at 60 °C for 6 h to evaporate the residual DMF solvent after the electrospinning process.

### 2.3. Polymer Solutions and Films: Rheological and Microscopy Analysis

The polymer blends’ rheological behavior was determined at room temperature (25 °C) using flow and frequency sweep tests. These measurements were conducted using an Anton Paar MCR 302 modular compact rheometer equipped with a cone (25 mm, 1°) and plate fixture. Using flow sweep tests, the shear rate varied between 1–1000 s^−1^. Frequency sweep tests in oscillation mode were conducted using the same cone & plate geometry in the range of 100–0.1 rad/s (strain amplitude 1%) at 25 °C. All measurements were collected within the linear viscoelastic regime (LVE) of each sample. The distribution of LSL and AL nanofibers within the PAN-MA polymer solution were macroscopically analyzed with polarized optical microscopy using a polarized light microscope (Zeiss AX10 with Axio Cam HRc). To analyze lignin distribution within dried PAN-MA/lignin films, a film solvent casting approach was used. The films were prepared by placing each polymer solution on a glass microscope slide followed by solvent evaporation at 60 °C for 12 h in an oven. The films were examined with the same microscope and also with a scanning electron microscope (SEM; Phenom ProX, Nanoscience Instruments, Eindhoven, The Netherlands) with an operating voltage of 5 kV or 15 kV.

### 2.4. Morphological, Chemical, and Thermal Characterization of Electrospun Nanofibers

The electrospun nanofibers’ morphology and diameter were examined using scanning electron microscopy (SEM; Phenom ProX) with an operating voltage of 15 kV and a high-resolution SEM (Hitachi S-4800) at an operating voltage of 30 kV. The mean diameter and standard deviation of the nanofiber thickness were estimated from the average of at least 50 measurements using ImageJ software. Attenuated Total Reflectance Fourier Transform Infrared (ATR-FTIR) spectra were collected using a Nicolet iS50 instrument (Thermo Scientific). A minimum of 64 scans were collected for all spectra recorded in the 4000–500 cm^−1^ range using a Smart Golden Gate ZnSe ATR accessory. The thermal stability of each sample was evaluated using Thermogravimetric analysis (TGA; Q500-0845-TGA model, TA Instruments). Thermograms were obtained using 4 mg samples heated on a platinum pan at a heating rate of 10 °C/min from 30 to 800 °C under a nitrogen atmosphere. Each thermogram was normalized with 100% weight percentage at ~130 °C to remove solvent effects (i.e., DMF residues or adsorbed water onto the nanofibers). Thermal transitions—glass transition temperature (Tg), melting temperature (Tm) and crystallization temperature (Tc)—were identified for each electrospun nanofiber via Differential Scanning Calorimetry (DSC) and Modulated Differential Scanning Calorimetry (mDSC) experiments using a Q2000 instrument (TA Instruments). Samples were placed in hermetically sealed T-zero low mass aluminum pans for DSC and mDSC measurements with sample weights between 1–3.5 mg. DSC measurements were made while heating each sample from 40 to 400 °C with a heating rate of 10 °C/min while mDSC measurements were performed at 2°/min, 1 °C amplitude, and 60 s periods up to 200 °C. All tests were performed under an inert nitrogen atmosphere.

## 3. Results and Discussion

**Figure 1:**
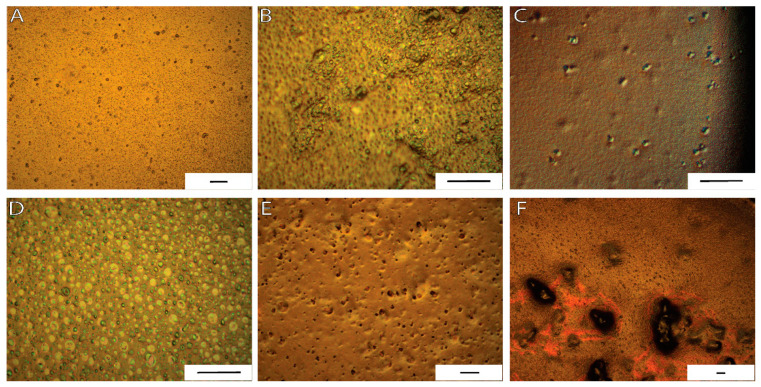
Polarized optical light micrographs of solvent casted polymer films: (**A**) AL-1; (**B**) AL-2; (**C**) AL-3; (**D**) AL-4, (**E**) LSL-1; and (**F**) LSL-2, revealed phase separation and aggregation of lignin within PAN-co-MA. [Scale bar = 50 microns].

PAN-MA/lignin polymer weight ratios were selected for electrospinning after a thorough microscopy inspection of solvent cast films (Table 1). In a previous literature study, solvent cast films provided detailed information about the interactions of polymer blends, or composites, prepared with lignin in a polymer matrix [39]. In the current study, the PAN-MA/lignin films were prepared in an effort to assess the effects of the lignin type and concentration on the distribution, phase separation, orientation, or aggregation of lignin within PAN-MA. As expected, the neat PAN-MA solvent cast film had a smooth surface without significant defects or roughness (Figure S1). Through the addition of lignin to PAN-MA, a birefringence response was expected for the films under polarized light, as reported elsewhere [36]. AL containing sample micrographs showed well-dispersed, round lignin aggregates (~5 micrometer diameter) within PAN-MA at a PAN-MA/AL ratio of 80/20 as shown in Figure 1A for 10 wt % total polymer (AL-1 sample) and in Figure 1C for a 12 wt % sample total polymer (AL-3 sample). At a 50/50 PAN-MA/AL ratio (AL-2 and AL-4), lignin birefringence was more evident within PAN-MA films and the formation of a porous structure was noted (Figure 1B,D). When the total polymer wt % increased from 10 wt % (AL-2) to 20 wt % (AL-4), the lignin aggregate size increased. Despite the rough surface observed at 50/50 blend ratios, AL seems to have distributed homogeneously within PAN-MA independent of the total polymer concentration of 10 wt % or 20 wt %. It is important to note that the PAN-MA/AL polymer solutions were magnetically stirred for only 2 h, as discussed in the Methods section.

Preliminary PAN-MA/LSL solvent evaporated films were obtained; however, phase separation was observed for short mixing times. Upon further inspection, an overnight mixing step (12 h) was implemented to improve the low-sulfonate lignin’s dispersion within the PAN-MA films. The additional mixing time significantly improved lignin dispersion for an 80/20 PAN-MA/LSL content, as shown in Figure 1E (LSL-1). As the LSL content increased from 20% to 50%, large aggregates formation continued to be observed within the PAN-MA films shown in Figure 1E (LSL-2). For this reason, both LSL samples were only produced at a total polymer content of 10 wt % in this study. Further increases in LSL ratio or total polymer content (e.g., 20 wt %) was unfeasible as more aggregates were prone to form. SEM micrographs were collected to further confirm the presence of lignin agglomerated distribution within the PAN-MA films and film morphology (Figure S2). SEM microscopy confirmed that LSL samples showed larger cluster formation, flaky structures, and large pores in contrast to AL containing samples.

**Figure 2:**
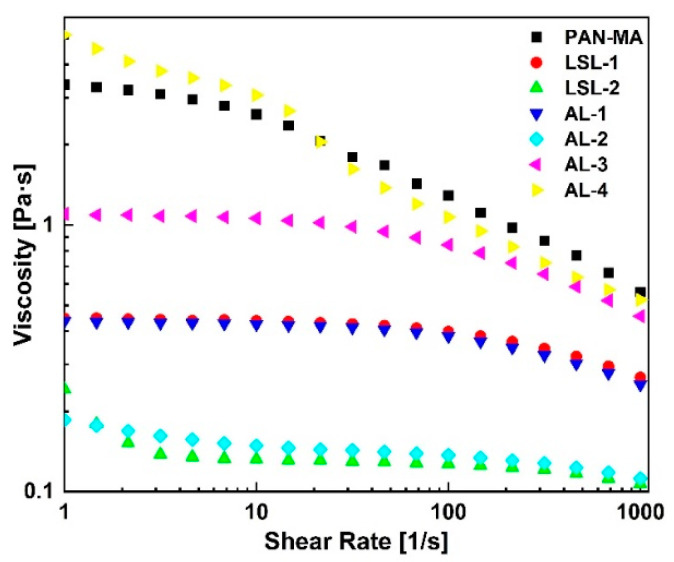
Steady-flow rheology results of all polymer solutions revealed that viscosity can be tuned based on the lignin content and the total polymer concentration. [PAN-MA in DMF (squares) was prepared at a 10 wt % in DMF and used as a control sample].

The viscosity results for the PAN-MA/lignin solutions are shown in Figure 2. The curves illustrate the shear-thinning behavior of the polymer solutions which was attributed to the macromolecular nature of the polymers [40]. At a 10 wt % polymer concentration, the control sample (pure PAN-MA/DMF solution, no lignin) exhibited the highest viscosity due to a ten-fold high molecular weight of PAN-MA relative to lignin. The viscosity steadily decreased as the lignin content increased in the polymer blend solution (compare LSL-1 to LSL-2 and AL-1 to AL-2). Note that, for the same PAN-MA/lignin ratios, the lignin type did not affect the viscosity results. For example, LSL-1 and AL-1 showed similar viscosity values. However, variations in viscosity were observed as the total polymer concentration in solution increased to 12 wt % and 20 wt % (see AL-3 and AL-4). AL-3 contained slightly less PAN-MA in solution as the control sample (9.6 wt % vs. 10 wt %) in addition to 2.4 wt % AL, but the viscosity was lower than the control sample. AL-4 contained the same amount of PAN-MA in solution as the control (10 wt %) but also contained 10 wt % AL. Its viscosity curve was essentially the same as the control despite containing alkali lignin. These results imply that the viscosity of the solution is dominated by the PAN-MA concentration, and the amount of lignin is inconsequential. This behavior implies that the high molecular weight PAN-MA serves as a type of scaffold in solution, which carries the low molecular weight lignin. This study confirms that lignin biopolymers can be added at various concentrations of interest without impacting the solution viscosity as long as the PAN-MA concentration is fixed.

Previous studies suggested that the electrospinnability of polymer solutions, comprised of lignin, depends on the viscosity behavior of the polymer solution, which dictates the nanofibers’ morphological properties [41]. Furthermore, PAN-MA and 80/20 PAN-MA/lignin blend viscosity curves exhibited a Newtonian plateau from low shear rate up to at least 10 s^−1^ (LSL1, AL-1, AL-3; Figure 2). Note that larger lignin ratios (50/50) showed a slight increase in viscosities at low shear rates; however, this was probably due to the rheometer reaching the lower limit of torque and was not considered significant. Thus, all samples were safely within their zero-shear viscosity range under 1 s^−1^ with the exception of PAN-MA. The shear rate in the electrospinning needle, estimated according to laminar flow in a capillary (shear rate = 4 Q/p R3), was approaching 0.1 s^−1^; therefore, the flow behavior during electrospinning was considered to be Newtonian for all samples. Frequency sweep tests in oscillation mode confirmed the polymer solutions’ liquid-like behavior at different lignin concentrations and total polymer concentrations. At angular frequencies below 100 rad/s, G’’ > G’ for all the samples (Figure S3).

**Figure 3:**
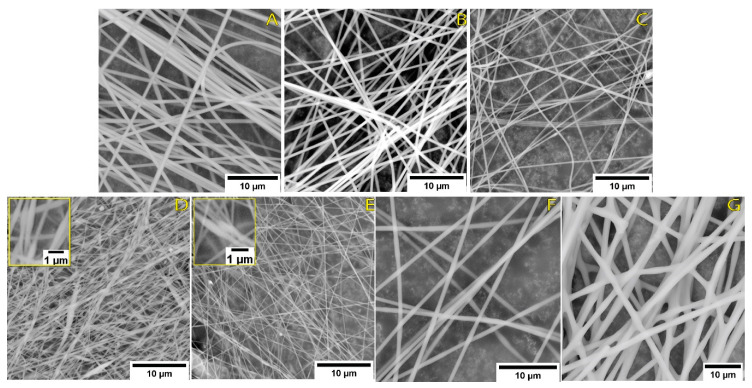
SEM images of electrospun PAN-MA/lignin nanofibers at a total polymer concentration of 10 wt % for (**A**) neat PAN-MA (control), (**B**) AL-1, (**C**) LSL-1, (**D**) AL-2, and (**E**) LSL-2; and at (**F**) 12 wt % AL-3, and (**G**) 20 wt % AL-4.

**Figure 4:**
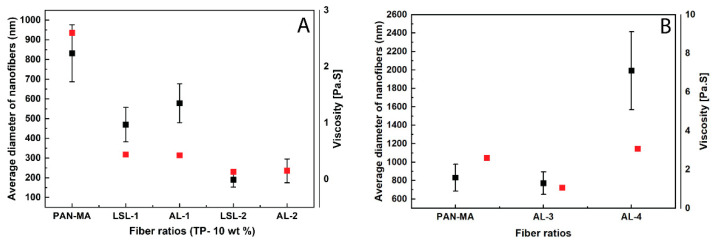
Correlations obtained between PAN-MA/lignin electrospun nanofibers’ diameter with polymer solution viscosity at 10 s^−1^ for (**A**) fibers of different lignin types and blend ratios at a total 10 wt % and (**B**) fibers at higher polymer content of 12 wt % (AL-3) and 20 wt % (AL-4). [Points with error bars represent nanofiber diameters].

The control sample PAN-MA (PAN-MA in DMF solution at 10 wt %) exhibited the highest viscosity of all samples and resulted in a stable jet during electrospinning and defect-free continuous nanofiber mats as shown in Figure 3A. Additionally, these fibers had the largest average diameter for the samples prepared at a 10 wt % polymer content (Figure 4A). AL-1 and LSL-1 solutions also showed a continuous fiber morphology (Figure 3B,C), and their average diameters were slightly less than PAN-MA. A high-resolution SEM image of AL-1 demonstrated a smooth electrospun nanofiber surface (Figure S4). When the ratio of LSL or AL to PAN-MA was increased to 50/50 (LSL-2 and AL-2), the fiber morphology resulted in nodules, or beads, along its length (Figure 3D,E) and their average diameters were the lowest of all samples (Figure 4A). Moreover, LSL-2 and AL-2 coincided with the lowest solution viscosities of all samples tested. During electrospinning, these samples were observed to form droplets at the needle tip, and the process gradually electrosprayed with improper formation of fibers (Figure 3D,E insets). A few droplets and aggregated structures were observed within the electrospun nanofibers, in particular for the LSL samples, confirming the aggregation phenomena observed in films produced via solvent casting (Figure S5A).

As the polymer content increased, AL-3 and AL-4 fibers were observed to have a continuous, smooth fiber surface (Figure 3F,G), referred to in some literature studies as “beads-free” morphology [36]. For AL-4 fibers, inter-fiber bonding was present with fiber diameters in the micron-sized scale (Figure 4B and Figure S5B); thus, the fiber-like structure is also affected by an increase in AL lignin content. In this study, a polymer solution viscosity range of approximately 0.45–3.5 Pa-s was observed to be a threshold of achieving beads-free morphology in the current study. Currently, whether the viscosity results are coincidental or directly related is unknown. The phenomenon of how the nodular (bead) morphology is obtained is not well understood in the literature, but the results of this work suggest that bead formation is related to lignin phase separation within PAN-MA. For instance, PAN-MA nanofibers exposed to polarized light revealed a single polymer phase (Figure S6A). Polarized micrographs of electrospun PAN-MA/lignin fibers revealed discontinuities within AL samples suggesting phase separation of the two polymers (Figure S6B,C). Although the polymer solution rheology was expected to impact electrospun nanofiber formation, the difference in solution viscosities between the produced beaded fibers and beads-free fibers was not significant (Figure 4, red squares). Moreover, electrospun nanofiber average diameters were significantly different for almost all the electrospun fibers (alpha = 0.05, Figure S7, One-way ANOVA, Tukey’s test). We only found non-significant differences in average diameter for three samples: PAN-MA and AL-3, LSL-1 and AL-1, and LSL-2 and AL-2. Additional factors experimentally studied in the literature include electrical conductivity, applied voltage, and surface tension [42]. Future studies could also correlate additional polymer solution parameters, such as electrical conductivity and surface tension [32].

In the industrial processes of polymer extrusion and fiber spinning, complex fiber morphologies such as sharkskin and melt fracture have been observed for pure polymers extruded at high shear rates, in some cases as low as 69 s^−1^ for the appearance of sharkskin [43]. The phenomenon has been closely linked to normal forces in the melt [44] and high shear stress at the wall, with critical value being on the order of 0.1 to 0.4 MPa [45]. However, the fiber morphology in the current study is not likely related to this due to the low shear rates, solution phase extrudate, and the presence of other factors related to electrical conductivity and rapid evaporation. What can be said for the current student is that, at the same total polymer content (10%), the average electrospun nanofibers’ diameters decreased as the lignin content increased, which corresponded to lower viscosity values (Figure 4A). As the total polymer content increased, at a fixed PAN-MA concentration, the nanofiber thickness and viscosity of the solution also increased (Figure 4B).

**Figure 5:**
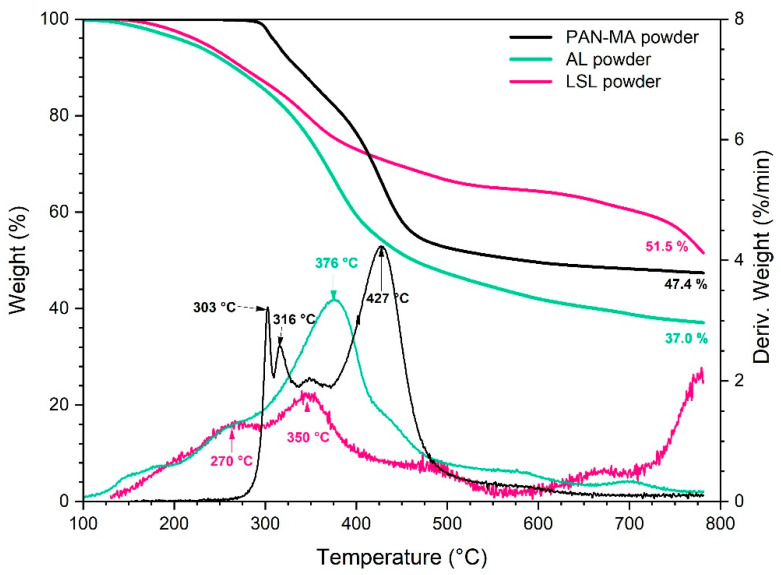
TGA results for powder raw materials (10 °C/min, nitrogen).

The purpose of TGA testing was to evaluate the decomposition behavior of the various powders and electrospun nanofiber materials, with a focus on how the material converts to a char which is important for the production of carbon fibers. First, the individual responses of the three raw materials in powder form are given in Figure 5 along with the wt % remaining curves and derivative curves. PAN-MA exhibited three primary stages (peaks at 303, 316, 427 °C) and a char yield of 47.4 wt %. This is reasonably consistent with other published studies that revealed PAN-MA undergoes degradation of the C-N bond at 297.5 °C [39]. In contrast, AL and LSL decomposition began a broad decomposition event around 150 °C. This is also consistent with previous studies, in which this effect was attributed to aromatic groups present in lignin and various molecular interactions between the bonds that are responsible for an extensive range of degradation [46]. The decomposition of AL powder was essentially single stage with a peak rate loss at 376 °C and a char yield of 37.0 wt %. LSL powder exhibited two stages (270, 350 °C) and a fairly high wt % plateau until after 750 °C when the weight remaining dropped to 51.5 wt %.

**Figure 6:**
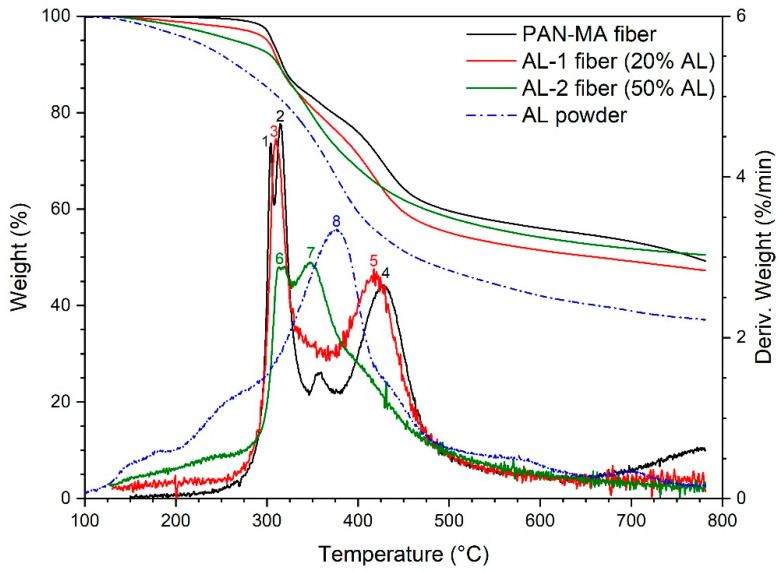
TGA results for AL-containing nanofibers as compared to pure PAN-MA fibers. Numbers denote points of discussion in the text.

The TGA results for AL-containing electrospun nanofibers are given in Figure 6. The results for the control fiber (PAN-MA) were comparable to the PAN-MA powder, with derivative peaks (points 1, 2, 4) located at the same temperature as in Figure 5. With the addition of 20% AL, the first two derivative peaks for PAN-MA (points 1,2) condensed into one (point 3), and the higher temperature peak (point 4) shifted to slightly lower temperature (point 5). No new peaks appeared (for example at 376 °C), perhaps because MA was present in such a low quantity. However, with the addition of 50% AL, a new derivative peak appeared around 350 °C (point 7), which may be attributed to the main AL decomposition (point 8) shift to a lower temperature. The peak corresponding to point 6 was attributed to PAN-MA decomposition, as was the case in the AL-1 fiber (i.e., point 3). The main conclusion drawn from analyzing the derivative peaks is that PAN-MA and AL do not decompose independently when blended together. This is not usual for polymer blends, in which case three possibilities exist that is difficult or impossible to predict: one of the components increases the stability of the other, one of the components reduces the stability of the components, or the blend has equal stability as the individual components [47]. Perhaps the most striking result of the AL containing fibers is that they produced a higher char yield than the AL powder and approximately the same char yield as the PAN-MA fibers. This positive synergy was considered to be a good result.

**Figure 7:**
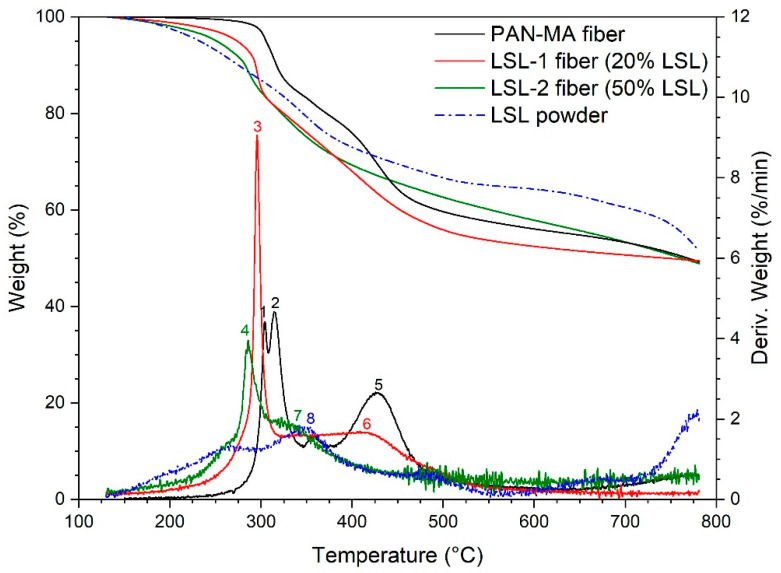
TGA results for LSL-containing nanofibers as compared to pure PAN-MA fibers. Numbers denote points of discussion in the text.

The TGA results for LSL-containing fibers are shown in Figure 7. With the addition of 20% LSL, the first two derivative peaks for PAN-MA (points 1,2) condensed into one (point 3) at slightly lower temperature, and the higher temperature peak (point 5) shifted to slightly lower temperature (point 6). With the addition of 50% LSL, the low temperature peak from LSL-2 shifted further to the left (lower temperature, compare point 4 to 3). In addition, the high temperature PAN-MA peak (point 5) does not appear in the LSL-2 response, but the 350 °C peak observed for LSL powder (point 8) does appear as a shoulder in LSL-2. The derivative peak results are similar to AL-containing fibers as seen in Figure 6. However, the net effect on wt % remaining was opposite, where the addition of LSL reduced the level of the high temperature plateau observed in LSL powder between 500–750 °C. On the other hand, the late drop in mass recorded for LSL powder (750–780 °C) placed all four materials in a similar range of final char yield.

A comparison of the weight loss curves for all five fibers is offered in Figure S8. Despite the fact that the low temperature weight loss behavior (for example, onset of weight loss) was significantly different, the final char yields at 780 °C were all in a similar range. The average char yield was about 49%. However, it is interesting that AL-2 fiber had the highest value (50.5%), while AL-1 fiber had the lowest (47.3%). Additional research would be needed to determine the statistical significance of these differences. Additionally, two of the materials (PAN-MA, LSL-2) lost weight at a higher rate than others at the cut-off temperature of 780 °C, so additional insight could be gained by investigating a higher final temperature, perhaps in the 800–900 °C range.

**Figure 8:**
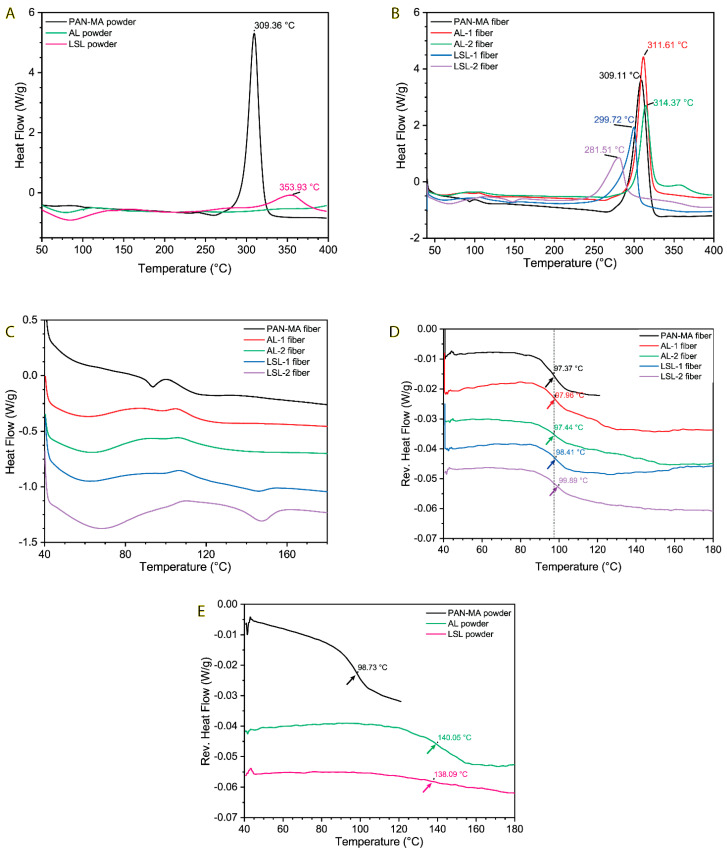
(**A**) DSC results for powder samples, (**B**) DSC results for fiber samples, (**C**) close−up of DSC results for fiber samples in Tg range, illustrating difficulty of assigning Tg values, (**D**) MDSC results for fiber samples, with clear glass transitions, and (**E**) MDSC results for powder samples.

Differential Scanning Calorimetry (DSC) was used to further study the decomposition behavior of the blends at the relatively low end of the spectrum (T < 400 °C), as well as to measure the glass transition temperature of the blends (Table 2). Generally, the T_g_ value(s) for a polymer blend can often be used to infer information about the phase behavior, i.e., whether the blend is homogeneous (one T_g_ value) or heterogeneous (two T_g_ values). The DSC results for the powders are given in Figure 8A. PAN-MA powder shows an exothermic peak at 309 °C, in agreement with the onset decomposition peaks from the TGA data observed in Figure 5. This sharp peak is consistent with nitrile group cyclization [48]. The peak at 353.9 °C for LSL powder is also consistent with TGA results from Figure 5. Interestingly, AL powder did not exhibit a DSC peak at 376 °C, although the curve appears to be trending upward as it approached 400 °C. At lower temperatures, the AL and LSL powder curves both show a broad endotherm between 40–120 °C. With further investigation, this was attributed to the evaporation of water absorbed in the polymer (approximately 5 wt %).

DSC results for the electrospun fibers are given in Figure 8C. The main decomposition peak of PAN-MA fiber is the same as the powder (309 °C). When PAN-MA was blended with increasing amounts of AL, this peak temperature increased, while PAN-MA blended with LSL resulted in a decreasing peak temperature. This trend is consistent with the TGA results given in Figure 6 and Figure 7. To more closely evaluate T_g_ results, the curves were enlarged and offset vertically to give Figure 8C. The irregularity of these curves made it impossible to determine the T_g_ of each material. Therefore, modulated DSC (mDSC) was conducted to help resolve these values, as given in Figure 8D. These results show that all the fibers exhibited a clear T_g_ around 97 °C. There was no apparent shift with the AL-containing fibers relative to pure PAN-MA, while the LSL containing fibers exhibited a 1 or 2 °C shift to higher temperatures (although this may be within the uncertainty of the measurement). Surprisingly, no indication of a glass transition was observed for the MA or LSL component of the blend, which was expected to occur around 140 °C based on the results of the pure powders (Figure 8E). The possible exception was for the AL-2 fiber (50% AL) in which it may be possible to interpret a broad step down between 110–160 °C. The T_g_ event for the PAN-MA powder and AL powder were of similar strength, while the LSL powder T_g_ was weaker and somewhat broader. The T_g_ results, while interesting, were not sufficient to allow us to make conclusions on the heterogeneity of the fibers.

**Figure 9:**
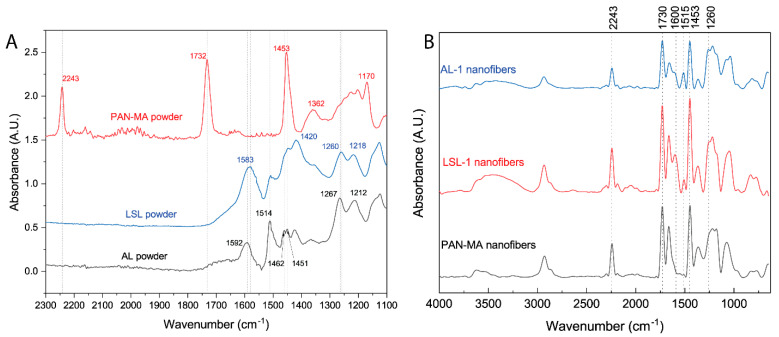
FTIR spectra of (**A**) LSL, AL, and PAN−MA (control) powders and (**B**) electrospun nanofibers of PAN−MA fibers (control), LSL−1 and AL−1. Both lignin samples had the same PAN−MA/lignin blend ratio (80/20) and were prepared at a 10 wt % concentration in DMF.

The chemical characterization of the polymer powders and electrospun nanofibers were performed using ATR-FTIR. The FTIR spectrum for a PAN-MA powder sample shows characteristic stretching vibration for -C≡N at 2243 cm^−1^, C=O at 1732 cm^−1^, and C-O at 1170 cm^−1^ (Figure 9A) [49]. These vibrations were not observed in either lignin powders (Figure 9A; LSL and AL). Within the fingerprint region, the LSL powder sample spectrum shows peaks at 1583, 1514, and 1420 cm^−1^, which were assigned to aromatic skeletal vibrations [33,50]. Similar peaks were also found for AL powders at 1583, 1514, and 1420 cm^−1^. Two additional bands characteristic of lignin were observed for LSL powders at 1260 cm^−1^ for C-O stretching and at 1218 cm^−1^ for C-C/C=O/C-O [50]. Slight shifts were also found for AL powder samples to 1267 cm^−1^ and 1218 cm^−1^. These slight differences between LSL and AL powders could result from the desulfonation process, required to obtain low sulfonate lignin powders [51]. A complete IR spectra for all powders can be found in Supplemental Figure S9.

FTIR analyses of PAN-MA/lignin electrospun nanofibers were undertaken to confirm the presence of lignin and to gain insights into lignin and PAN-MA interactions. Figure 9B displays the IR spectra for the 80/20 PAN-MA lignin fibers prepared at a 10% total polymer content (AL-1 and LSL-1). Additionally, an IR spectrum for a PAN-MA nanofibers control sample is shown. As expected, all electrospun PAN-MA/lignin fibers possess the characteristic PAN-MA peaks (2243 cm^−1^, 1732 cm^−1^, and 1170 cm^−1^). Broad -OH (~3300 cm^−1^) and CH_2_ stretching (2930 cm^−1^) peaks are typically found in lignin samples [52], and were observed in LSL-1 and AL-1 but not in PAN-MA (Figure 9B). These results confirm the presence of lignin within PAN-MA/lignin blends’ electrospun nanofibers. Note that the PAN-MA/lignin blends show peak shifting as compared to neat powder samples. For instance, the carbonyl stretching vibration associated with aromatic rings was shifted to a higher wavenumber of ~1600 cm^−1^ for both electrospun lignin fibers (AL-1 and LSL-1). This shift is attributed to potential interactions between lignin and PAN-MA [53]. The C-O stretching peak at 1260 cm^−1^, characteristic for lignin, was also found in both PAN-MA/lignin electrospun nanofibers. Compared to the samples’ powder IR spectra, a red shift from 1267 cm^−1^ to 1260 cm^−1^ was observed only for the AL-1 blend; however, this shift was not observed for the LSL-1 sample. This finding supports that AL compatibility with PAN-MA is superior as compared to PAN-MA/LSL blends, confirming microscopy observations.

## 4. Conclusions

In this study, we demonstrated the successful production of electrospun nanofibers with the addition of alkali, Kraft lignin (AL) to PAN-MA at blend ratios of up to 50 wt % with a total polymer content up to 20 wt % in DMF solvent. These nanofibers exhibited PAN-MA/AL compatibility, a smooth morphology, and nanofiber diameters in the sub-micron scale region, as confirmed via microscopy and infrared spectroscopy measurements. As compared to neat PAN-MA, AL presence did not affect the fibers’ glass transition temperature (~97 °C) and did not affect the char yield (~47%) when heated in nitrogen to 800 °C. For PAN-MA/lignin blends, we found a minimum viscosity threshold of ~0.3 Pa-s to produce smooth electrospun nanofibers without a nodular morphology, independently of the lignin type. For LSL, however, we found poor compatibility with PAN-MA, which resulted in polymer phase separation, electrospun nanofibers with a nodular morphology, and polymer solutions prone to electrospraying. Ultimately, the degree of lignin sulfonation dictates the properties of high-quality lignin-based electrospun nanofibers. We conclude that PAN-MA/AL nanocomposite fibers could replace PAN or PAN-MA as precursors to producing carbon fibers or directly in other applications such as membranes, absorbents, supercapacitors, or electrical materials.

## Figures and Tables

**Table 1:** Weight ratios of PAN-MA:Lignin polymer blends prepared based on total polymer concentration in wt %.

PAN Content	Lignin Content	Total Polymer Content in wt %	Lignin Type	Sample ID
100	0	10		PAN-MA
80	20	10	Low sulfonate	LSL-1
50	50	10	Low sulfonate	LSL-2
80	20	10	Alkali, Kraft	AL-1
50	50	10	Alkali, Kraft	AL-2
80	20	12	Alkali, Kraft	AL-3
50	50	20	Alkali, Kraft	AL-4

**Table 2:** Glass transition temperature (T_g_) and decomposition temperature (T_d_) [endothermic peaks] of 10 wt % electrospun PAN-MA/lignin nanofibers.

Sample ID	T_g_, °C	T_d_ (Exo), °C
PAN-MA	97.4	309.1
AL-1	98.0	311.6
AL-2	97.4	314.4
LSL-1	98.4	299.7
LSL-2	99.9	281.5

## Data Availability

The data presented in this study are available on request from the corresponding author.

## Supplementary Materials

The following are available online at https://www.mdpi.com/2073-4360/13/7/992/s1, Figure S1: Polarized light optical microscopy image of PAN-*co*-MA dried films. Figure S2: SEM image of polymer solution blend film; (a) AL-1, (b) LSL-1, (c) AL-2 and (d) LSL-2. Inset in b and d shows enlarged LSL clusters at 10 µm. Insets in b and d show clusters formed at 10 µm. Figure S3: Frequency sweep data of storage modulus (G’) (black dots) and loss modulus (G”) (red dots) for PAN-MA/lignin blends, collected at a 1% strain amplitude. Figure S4: HR-SEM image of AL-1 nanofibers showing smooth structure. Figure S5: Low-magnification SEM images of LSL-2 and AL-4 samples show (A) large aggregates were present during the electrospinning of LSL-2 samples and (B) inter-fiber bonding was observed for sample AL-4. Figure S6: Polarized optical light microscopy images of electrospun nanofibers at 50 magnification for (a) PAN-MA; (b) AL-1; and (c) AL-3 samples. Figure S7: One-way ANOVA (Tukey’s) test to estimate significant differences for electrospun nanofiber average diameters based on SEM measurements. Figure S8: Compiled TGA weight loss results for electrospun fibers. Figure S9: Full FTIR spectra for AL-1, LSL-1, and PAN-MA control sample (referred to as PAN in the plot).
