# Supplementary material for: Fabrication and Characterization of Electrospun Poly(acrylonitrile-co-Methyl Acrylate)/Lignin Nanofibers: Effects of Lignin Type and Total Polymer Concentration
Source: Polymers (Basel). 2021 Mar 24;13(7):992. doi: 10.3390/polym13070992 (PMC8037837; doi:10.3390/polym13070992)
Supplement: Supplementary file 1 [file polymers-13-00992-s001.pdf]

SUPPORTING INFORMATION

Fabrication and Characterization of Electrospun Poly(acrylonitrile-*co*-Methyl Acrylate)/Lignin Nanofibers: Effects of Lignin Type and Total Polymer Concentration

Suchitha Devadas[1], Saja M. Nabat Al-Ajrash[1], Donald A. Klosterman[1], Kenya M. Crosson[2,3], Garry S. Crosson[4] and Erick S. Vasquez[1,3,*]

[1] Department of Chemical and Materials Engineering

[2] Department of Civil and Environmental Engineering and Engineering Mechanics

[3] Integrative Science and Engineering Center

[4] Department of Chemistry

University of Dayton, Dayton, OH, 45469,

* Correspondence: evasquez1@udayton.edu ; Tel.: +1(937)-229-2627

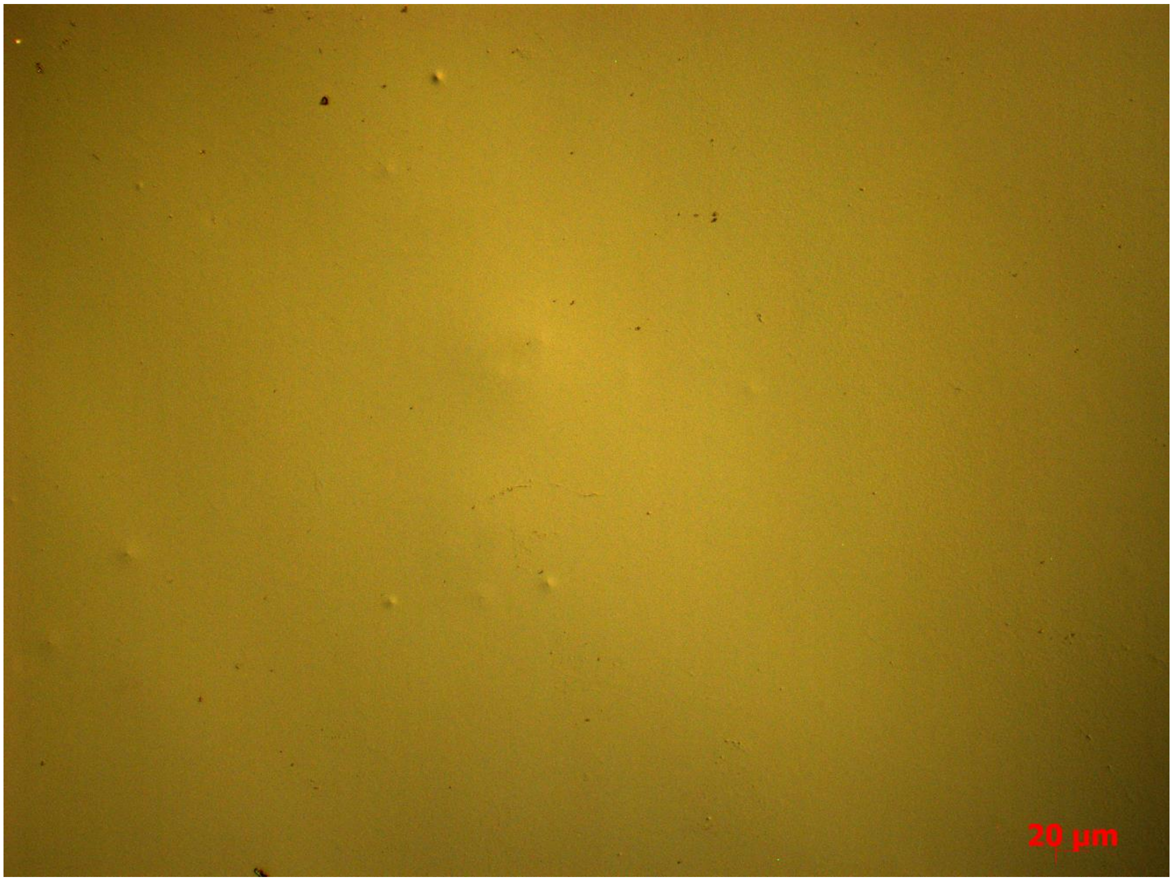

Figure S1: Polarized light optical microscopy image of PAN-*co*-MA dried films

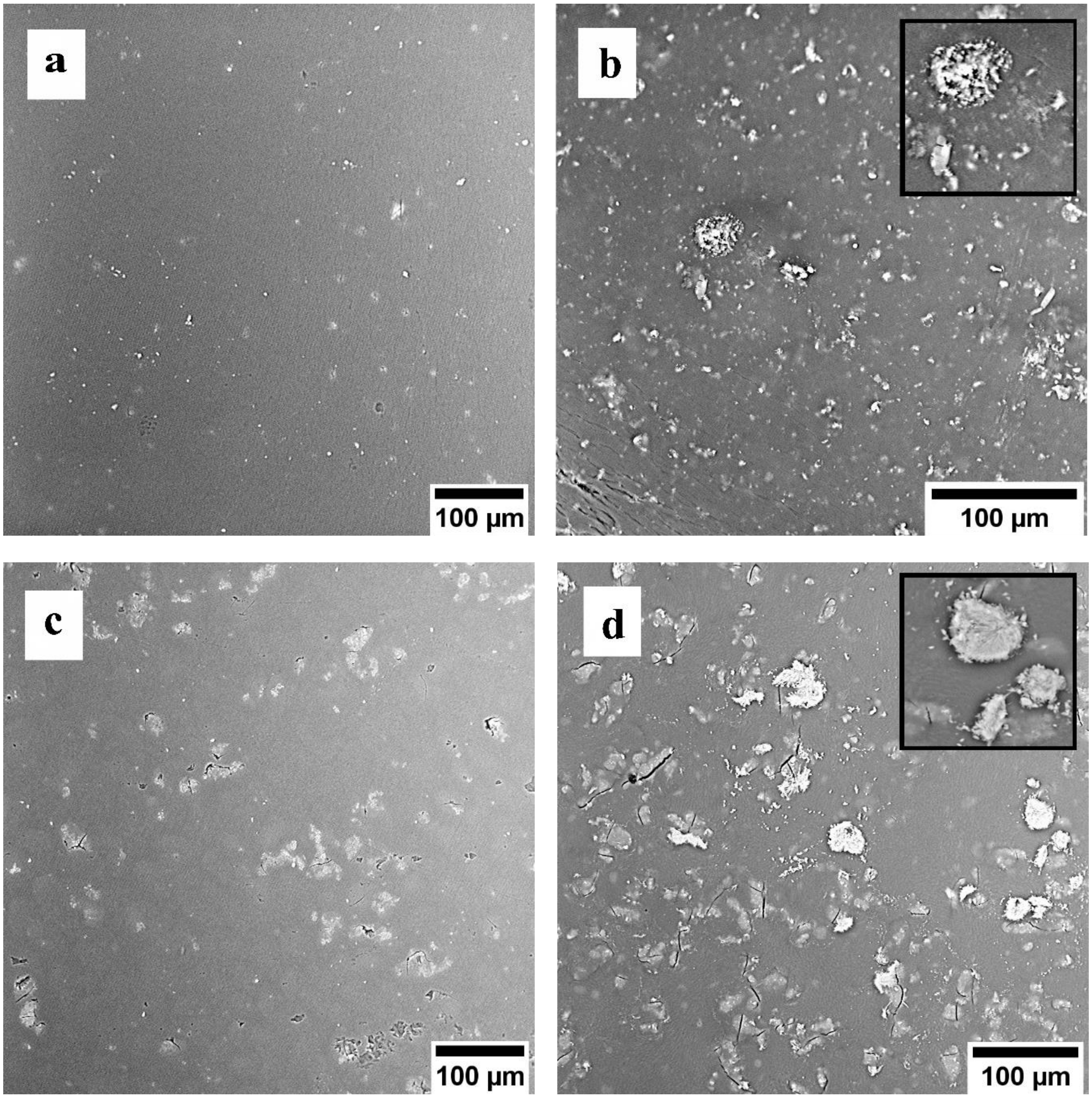

Figure S2: SEM image of polymer solution blend film; (a) AL-1, (b) LSL-1, (c) AL-2 and (d) LSL-2. Inset in b and d shows enlarged LSL clusters at 10 µm. Inset in b and d shows clusters formed at 10 µm.

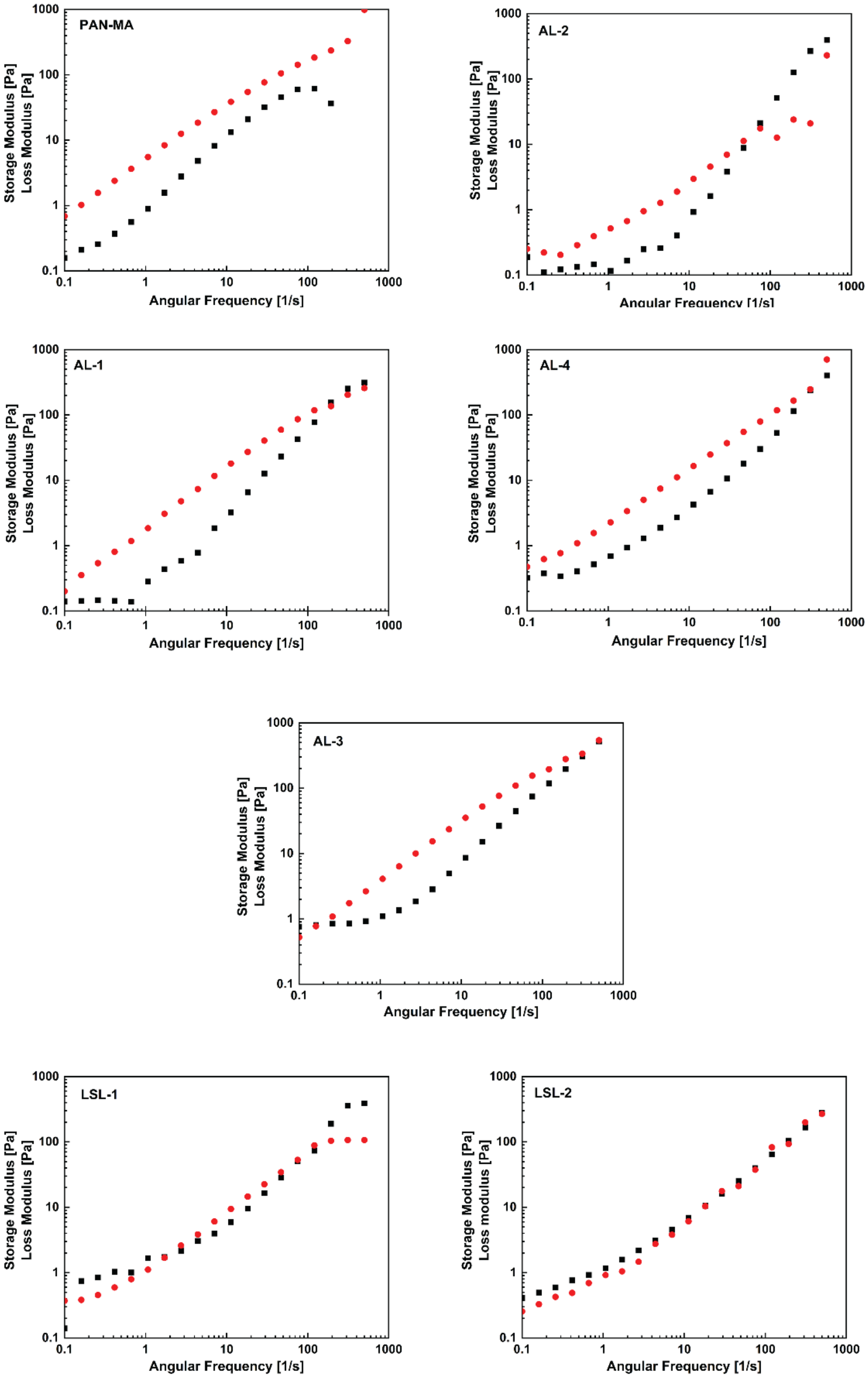

Figure S3: Frequency sweep data of storage modulus (G’) (black dots) and loss modulus (G”) (red dots) for PAN-MA/lignin blends, collected at a 1 % strain amplitude.

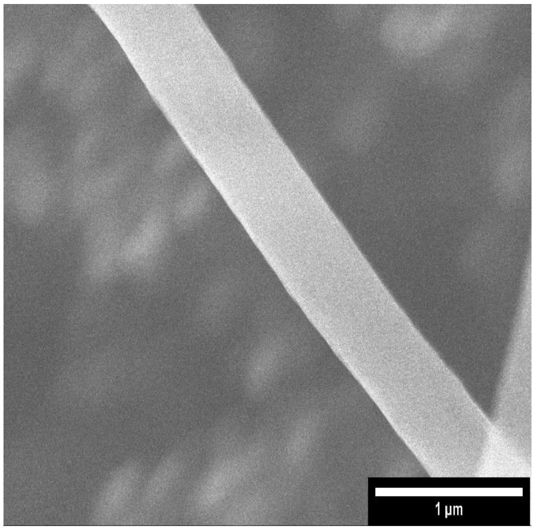

Figure S4: HR-SEM image of AL-1 nanofibers showing smooth structure.

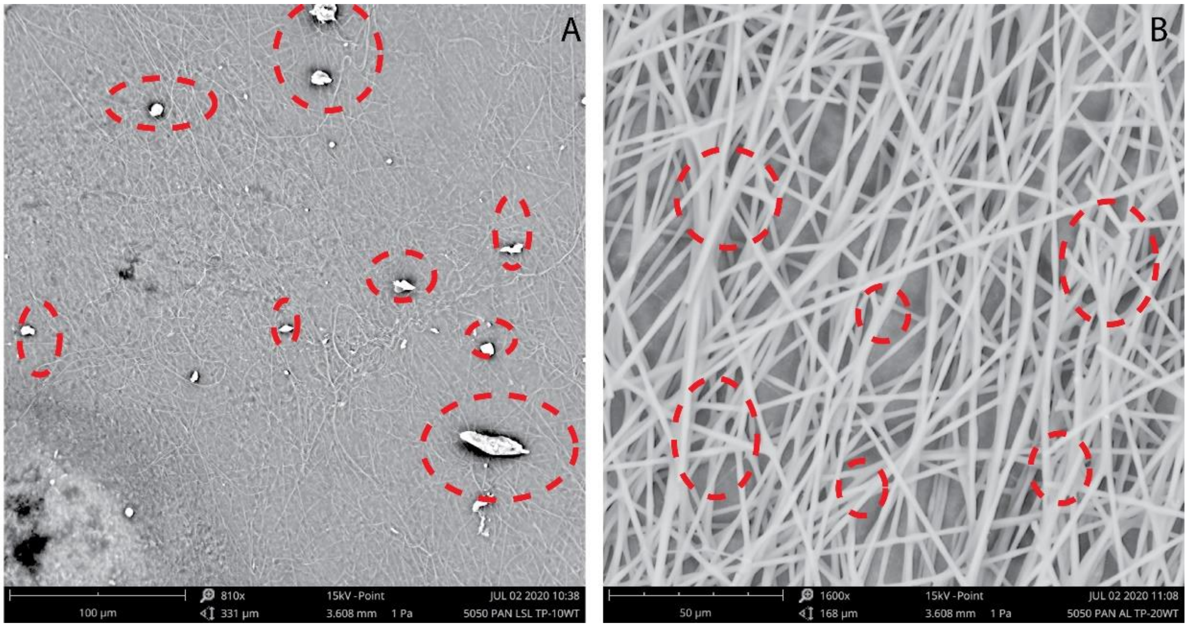

Figure S5: Low-magnification SEM images of LSL-2 and AL-4 samples show (A) large aggregates were present during the electrospinning of LSL-2 samples and (B) inter-fiber bonding was observed for sample AL-4

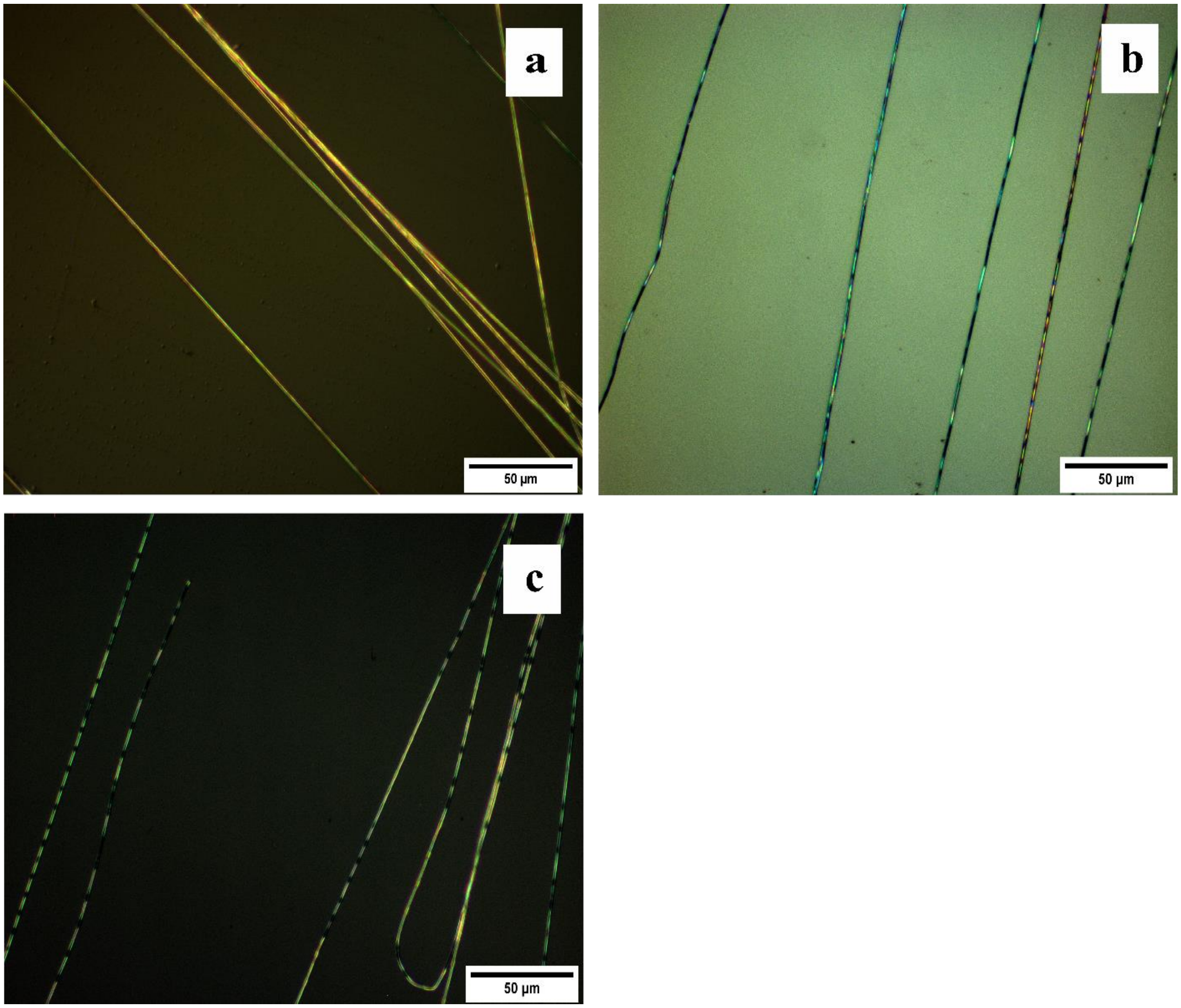

Figure S6: Polarized optical light microscopy images of electrospun nanofibers at 50 magnification for (a) PAN-MA; (b) AL-1; and (c) AL-3 samples.

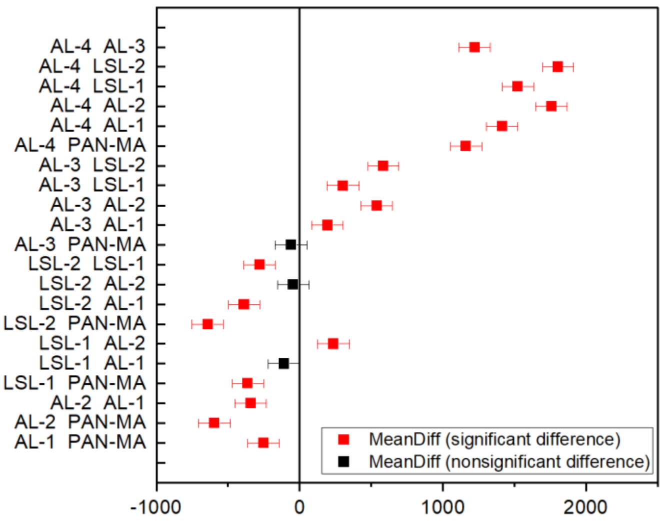

Figure S7: One-way ANOVA (Tukey's) test to estimate significant differences for electrospun nanofiber diameters based on SEM measurements

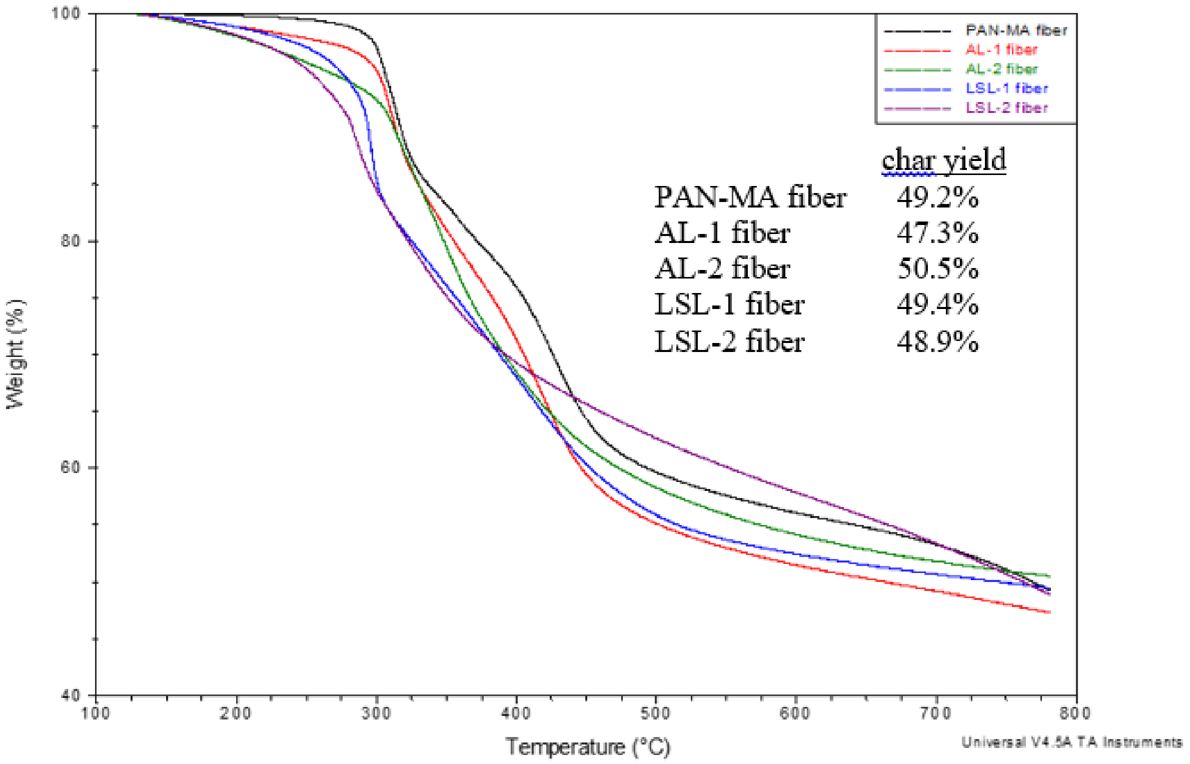

Figure S8: Compiled TGA weight loss results for electrospun fibers.

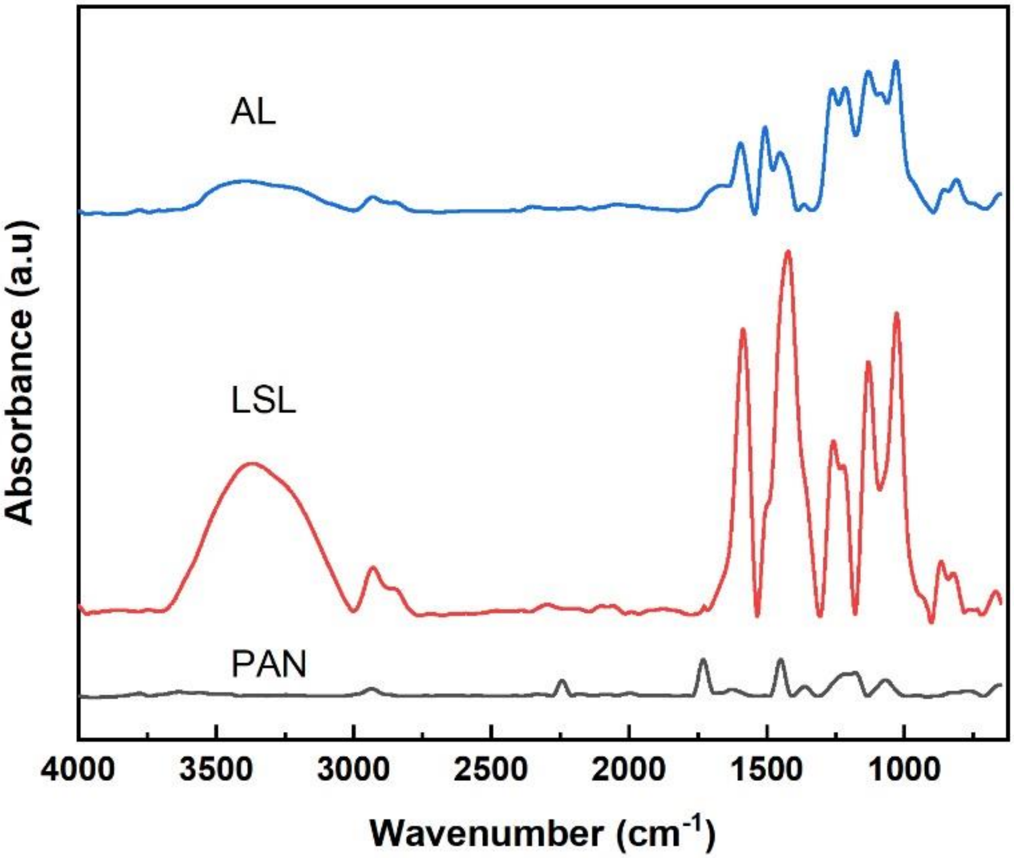

Figure S9: Full FTIR spectra for AL-1, LSL-1 and PAN-MA control sample (referred as PAN in the plot)
